# First person – Hung-Ju Chiang

**DOI:** 10.1242/dmm.050938

**Published:** 2024-07-16

## Abstract

First Person is a series of interviews with the first authors of a selection of papers published in Disease Models & Mechanisms, helping researchers promote themselves alongside their papers. Hung-Ju Chiang is first author on ‘ Male germ cell-associated kinase is required for axoneme formation during ciliogenesis in zebrafish photoreceptors’, published in DMM. Hung-Ju is a Junior Research Fellow in the lab of Ichiro Masai at Okinawa Institute of Science and Technology Graduate University, Japan, investigating the importance of cilia, a largely underappreciated organelle.


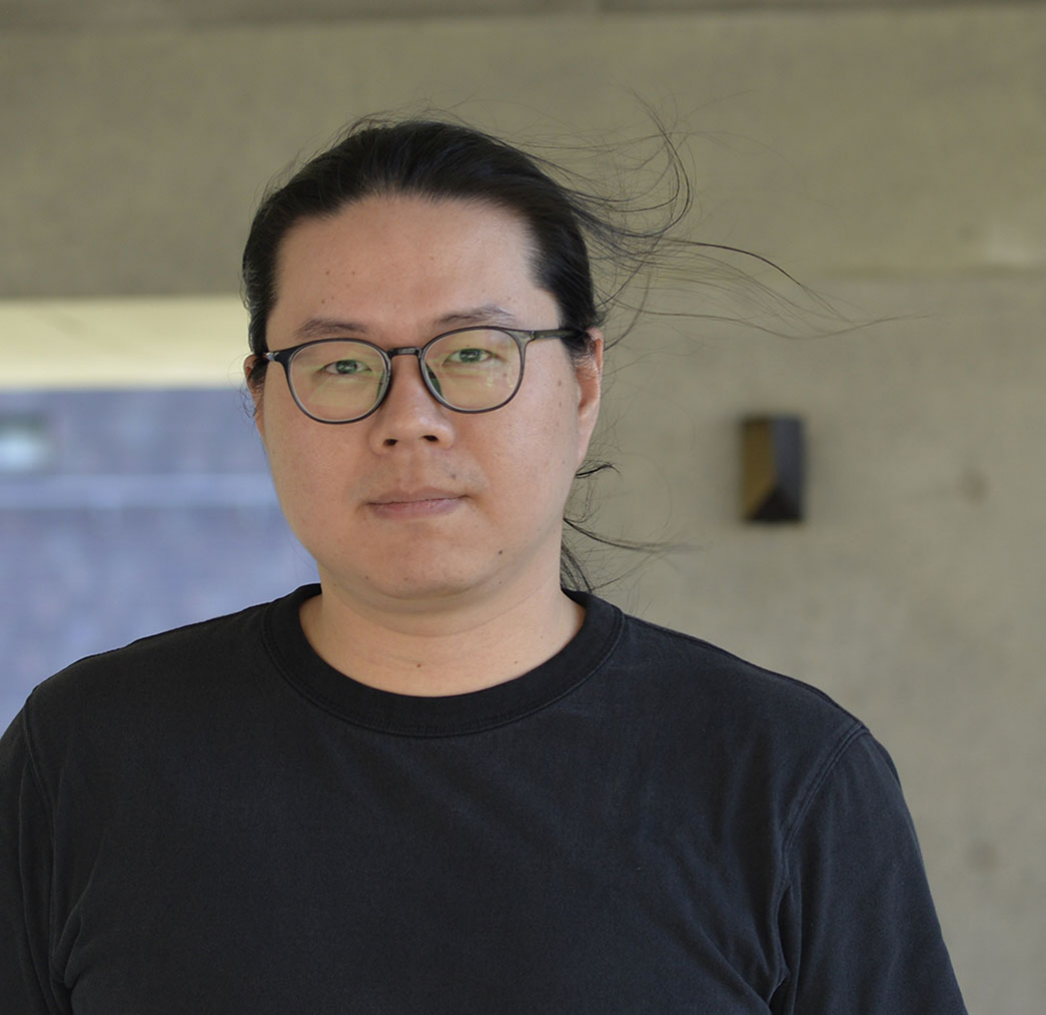


**Hung-Ju Chiang**

**Who or what inspired you to become a scientist?**

Since I was a kid, I have been curious about nature. I always tirelessly asked my parents questions about everything. Instead of telling me the answers, they encouraged me to find them myself. Later, I went to a medical school for college and interned in a hospital where I witnessed patients being diagnosed with diseases that had no cure. These experiences led me to the path of becoming a disease scientist.

**What is the main question or challenge in disease biology you are addressing in this paper? How did you go about investigating your question or challenge?**

MAK mutations cause photoreceptor degeneration and blindness in animals including humans, mice and zebrafish. Still, the reason the mutant photoreceptors degenerate and the role of MAK in photoreceptor physiology are not fully understood. In my research, I sought the answers by studying zebrafish *mak* mutant photoreceptors.

**How would you explain the main findings of your paper to non-scientific family and friends?**

Mutations of MAK lead to blindness, particularly photoreceptor degeneration, in humans, mice and zebrafish, but how it happens is not fully understood. In my work, I used *mak*-mutated zebrafish as a model to figure out the reasons behind the blindness. In *mak* zebrafish mutants, the light-sensing cells (photoreceptors) could not grow cilia, a tail-like structure that is essential for the formation of the light-sensing compartment (outer segments) of the cell, and this developmental failure resulted in cell death. In addition, I verified that Mak's enzymatic (kinase) function was important for a photoreceptor to grow a cilium. Without the kinase function of Mak, the photoreceptors cannot develop cilia and will eventually die. In conclusion, my work shows the importance of Mak in photoreceptor cilium formation and cell survival.

**What are the potential implications of these results for disease biology and the possible impact on patients?**

MAK deficiency causes an inherited blindness named retinitis pigmentosa, in which the patients' photoreceptors degenerate progressively. The mechanisms underlying the development of this disease are largely unknown. My results provide potential explanations for MAK-deficient photoreceptor degeneration and insights for further MAK-associated studies.

**Figure:**
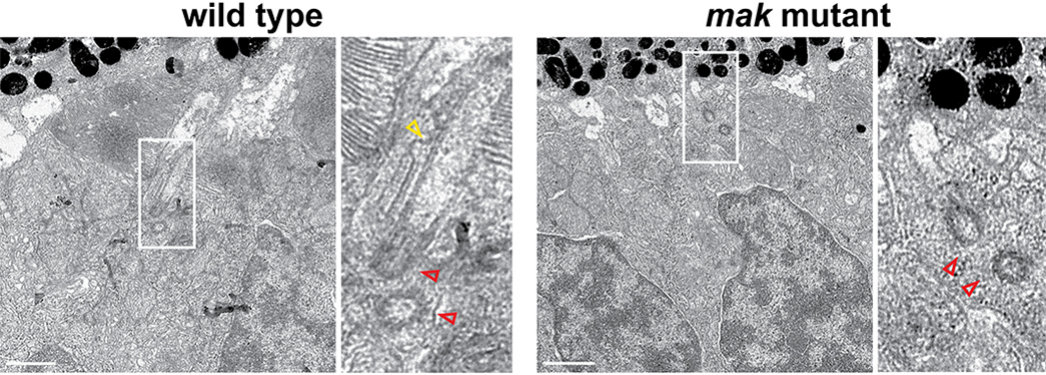
**Ciliary axoneme formation is disrupted in zebrafish *mak* mutant photoreceptors.** Red arrowheads, basal body; yellow arrowhead, axoneme. Scale bars: 1 µm.

**Why did you choose DMM for your paper?**

DMM focuses on advanced insight into disease biology, and it has a prestigious reputation and a history of publishing high-quality and impactful articles to improve understanding of disease processes, which aligns with our expectations for this paper.

**Given your current role, what challenges do you face and what changes could improve the professional lives of other scientists in this role?**

As a new PhD graduate, I noticed the importance of finding inner peace during my training. Life is stressful in academia. Countless times, I experienced anxiety, panic attacks and insomnia in the past few years, which affected my performance seriously. To deal with this, I started to practice meditation regularly, trying to figure out the fundamental problems and set solutions to them. The stress can be mitigated once a set of solutions is set.

**What's next for you?**

This work was the central theme of my PhD thesis, with which I was privileged to dive into the world of cilia. As a postdoctoral researcher in the next chapter, I will continue my journey in cilium biology/ciliopathy research.

**Tell us something interesting about yourself that wouldn't be on your CV**

I once struggled to make a career direction between two very different paths: I would have become a heavy metal drummer and music producer if I had not chosen to work on science.
